# Violence against women by male partners and against children within the family: prevalence, associated factors, and intergenerational transmission in Romania, a cross-sectional study

**DOI:** 10.1186/1471-2458-14-129

**Published:** 2014-02-07

**Authors:** Cornelia Rada

**Affiliations:** 1 Biomedical department, “Francisc I. Rainer” Anthropology Institute of the Romanian Academy, 8 Avenue Eroii Sanitari, O.P. 35, C.P. 13, Sector 5, 050474 Bucharest, Romania

**Keywords:** Domestic violence, Intimate partner violence, Violence against women, Witnessed parental violence, Child abuse, Psychological violence, Physical violence, Risk factors, Family

## Abstract

**Background:**

Domestic violence is a public health problem with negative consequences. We aimed to determine the prevalence of violence between parents and by parents against children, types of intimate partner violence against women, the intergenerational transmission of violence, and to identify a profile of beliefs and judgements regarding violent behaviour.

**Methods:**

The data used for this article were sourced from three cross-sectional studies performed in Romania in 2009–2011. We sampled 869 respondents (male and female) with a homogenous distribution between environment, gender, educational level, and age group (18 to 75). From a 96-item questionnaire regarding family and reproductive health, this article refers to four items: (1) feelings relating to the family in which they were raised; (2) whether they witnessed violence between parents or were victims of violence by parents or other family members during childhood or the teenage years; (3) opinions relating to 10 statements on violence from Maudsley Violence Questionnaire; (4) the manifestation of psychological, emotional, and sexual abuse from the partner in the family of procreation (FOP). The data were analysed by Pearson chi-square tests and latent class analysis.

**Results:**

During childhood, 35% of respondents witnessed parental violence and 53.7% were victims of family violence. Psychological abuse by men against women was the most common type of violence reported in the FOP (45.1%). Violence in childhood and adolescence correlated with the perception of the family of origin as a hostile environment and of violence against women as a corrective measure, and that insults, swearing, and humiliation by their partner within the FOP is acceptable (p < 0.05). A profile of beliefs and judgements about violent behaviour indicated that the Impulsive reactive cluster is represented by men in rural areas, and by subjects who witnessed parental violence or were victims of violence during childhood (p < 0.001).

**Conclusions:**

In Romania, the use of violence as a form of discipline or instruction of children and women remains a significant problem, with a higher rate of intimate partner violence than in other developed countries. Furthermore, implementing intervention mechanisms for psychological abuse is urgently required, as are education and intervention in high-risk populations.

## Background

The most widespread form of violence against women is intimate partner violence (IPV), which has negative consequences on the victims’ health [1,2]. If we consider domestic violence against children and spouses, the psychological consequences are also huge, stemming from the paradox of the victim being abused by a member of the family with whom he or she expects to have a supportive, loving, and respectful relationship [3-5].

IPV is considered a public health problem, and research worldwide has stressed the importance of its prevention and its negative effects on health [6]. Studies performed at both national and international levels have indicated that low levels of education and income are closely correlated to domestic violence, with the subjects being either witnesses or victims. These studies also indicated that violent behaviour may be transmitted across generations [7,8].

A number of factors appear to explain the varying prevalence rates for family violence among European countries: low economic living conditions, traditional gender attitudes towards women, authoritarian parenting, and a general tolerance of violent behaviour in a given society. In addition, a person’s ability to cope with a situation may influence whether one family member will act violently against another [9].

Explanations regarding the origins of IPV are based on a number of factors, including biosocial, psychological, and sociological. Children raised in homes where violence is used will learn to exercise violence in their own relationships, leading to a circle of violence. In addition, being on the receiving end of violence increases the risk of further violence—“violence begets violence” [10].

Some studies have described IPV within the context of patriarchal terrorism, stating that it is a result of a global model of power and control. Other studies have referred to IPV from a situational perspective and identified it as a result of a person’s incapacity to manage conflict efficiently [11,12]. International studies have indicated that 80%–98% of children worldwide have been physically punished within their family—a rate that is unacceptable. The real rate of violence is not known because of under-reporting [13,14].

### Domestic violence in Romania

A national-level study, the National Study on Domestic and Work Violence, was conducted in Romania in 2003, using a representative national sample of 1,206 men and women and a representative sample of 600 women; all were aged 18 years and older. The study found that 14% of the subjects in the national sample and 17% of the sampled women had experienced at least one form of abuse (emotional, psychological, physical, sexual, financial, or neglect) “on a frequent basis” in the family of origin (the family into which a person was born and grew up; FOO) or in the family of procreation (the family created through and following marriage or consensual union) (FOP). Almost half of the reported abuse was committed by the partners (male or female) of the respondents [15].

A national study in 2004, Reproductive Health Survey Romania 2004 (SSR-Ro 2004), included 15 items regarding violence against women and the violent behaviour of men. In the sample of 3,391 females (15–44 years), almost 30% reported that they had suffered from verbal, physical, or sexual abuse in the FOP. In the sample of 1,613 males (15–49 years), more than half reported that they conducted such behaviour on their partners in the FOP [16].

The proportion of women that were victims of violence and had also witnessed violence between their parents as children was more than double that of those who had not witnessed such violence. Women who come from a family with a history of parental violence, have been subject to violence from family members during childhood or adolescence, are unemployed, and with low educational levels are at a high risk of experiencing violence [17].

A further Romanian study performed in 2007, Domestic Violence in Romania: Legislation and the Judiciary System, estimated that half the women imprisoned for homicide were themselves victims of domestic violence and that most of them acted in self-defence. Thus, they are both culprits and victims [18].

It is only relatively recently—since 1995—that domestic violence has become the subject of public debate in Romania. Previously, the Romanian Criminal Code did not recognize domestic violence as an actual crime, and the power of authorities to intervene was limited. Despite changes in the law, the state and its executive bodies remain reluctant to intervene in domestic violence situations, believing these to be ‘private’ matters. The following is a brief summary of some of the laws and legislative stipulations that regulate domestic violence in Romania. Order No. 384/306/993 was issued in 2004, and it calls for collaboration towards preventing and monitoring domestic violence cases among the following three ministries: the Ministry of Labour, Family and Social Solidarity (now called the Ministry of Labour, Family and Social Protection); the Ministry of Administration and Interior; and the Ministry of Public Health. The most recent regulation was Law No. 25, which was passed in 2012; it is a modification and extension of Law No. 217, which was promulgated in 2003 to prevent and control domestic violence. Law No. 25 carries one essential modification: a woman abused within her family may request a court order for her protection and restrict further contact with the aggressor [19].

### Objectives

Relevant data on domestic violence are necessary for public health policies towards prevention and support. Given the above background, the main objectives of the present study were to determine the following: (1) the magnitude of domestic violence and the related socio-demographic factors with respect to violence between parents and by parents towards their children in the FOO; (2) the emotional states related to the FOO; (3) the magnitude and distribution of types of abuse against the wife or female partner in the FOP; (4) the extent to which being a witness to or the victim of domestic violence in the FOO is transmitted to the FOP; and (5) profiles of beliefs and judgements about violent behaviour according to gender, environment, and history of violence in the FOO by means of latent class analysis (LCA).

The merits of this paper are as follows. There are very few English-language studies on the family environment in Romania, and therefore this study fills this gap. This paper looks at ‘hidden’ violence in Romania, and in doing so, it focuses on the general public, i.e., those that may have not even considered domestic violence previously nor sought any assistance.

In addition, the study shows the correlation between the description of feelings towards the FOO and exposure to domestic violence within the FOP. Moreover, this paper demonstrates the association between violence in childhood and adolescence in the FOO and belief in the use of violence for corrective purpose.

## Methods

### Design and sampling

The data used for this article were sourced from three cross-sectional studies performed between 2009 and 2011 (see Acknowledgements). Although not representative of the entire country, the sample consisted of 869 randomly selected subjects (414 men and 455 women). The distribution of the subjects was relatively homogenous with respect to environment, gender, educational level, and age (Table 1).

**Table 1:** Distribution of subjects according to environment, gender, educational level and age group

**Sociodemographic variables**	**Number**	**%**
Environment		
Urban	435	50.1
Rural	434	49.9
Gender		
Male	414	47.6
Female	455	52.4
Educational level		
Low (primary education <10 years)	326	37.5
Medium (secondary education 10–12 years)	296	34.1
High^a^	247	28.4
Age groups (years)		
18–24	152	17.5
25–34	153	17.6
35–49	248	28.5
50–59	165	19.0
60–75	151	17.4

Legend: ^a^Tertiary education >12 years, completed by obtaining diploma, university, post-university.

The sample size was computed by taking into account the statistical tests and analysis to be conducted. G*Power software was used for the chi-square test, assuming an effect size of w = 0.5 and degree of freedom of df = 250; this produced a recommended sample size of 337 subjects by gender—a total of 674 men and women. Owing to the sensitivity of some questionnaire items, the expected missing data rate in the study was assumed to be 0.25; thus, the sample size was increased by a factor of 1.25 to 842 subjects. Accordingly, the study aimed to achieve a target conservative sample size of 850–900 subjects. A simple random selection of subjects was undertaken from the sampling frame.

For the sampling frame, we used lists obtained from local governments for the 2009 and 2011 studies (669 urban and rural subjects), and we contacted the individuals through local government officials, family doctors, and university rectors. For the 2010 study (200 urban subjects), we did not employ lists from local governments but used instead lists of patients from family doctors, lists of company employees, and lists of university students.

### Study setting

The study was conducted in urban areas within Bucharest, Craiova, and Satu Mare; it was also carried out in the villages of Cioroiaşi and Stolnici and in villages in the county of Satu Mare. When choosing these locations as statistical units, their sociodemographic and cultural characteristics were taken into account. These include the age of the settlement, population density, access by car, train and plane, and rank according to the plan for national territory arrangement. Thus, all the locations had their own unique features. Bucharest, Romania’s capital, has the country’s largest urban population; it is the nation’s educational centre and the hub for national and international road, rail, and air travel. Craiova, the administrative centre of Dolj county, is Romania’s fifth-most populous city, and it has important academic and cultural significance [20]. Cioroiaşi is a community (an administrative subdivision that contains several villages) in Dolj county, Oltenia; it is 45 km from Craiova and has a population of approximately 1,775 people in 953 households [21]. Stolnici is another community in Arges county, Wallachia; it is situated 38 km from the administrative centre of the county, the city of Pitesti. Stolnici village has approximately 1,505 inhabitants in 542 households [22]. Satu Mare county is located in north-west Romania, near the border with Hungary and Ukraine, and thus has high levels of temporary migration. It is also an area characterized by various religious and ethnic minorities [23,24].

The study subjects were not the clients of recovery centres for victims of domestic violence, which provide counselling, psychotherapy, or other services to victims. Whenever possible, a request was made that the partner of a respondent also completed the questionnaire. The statistical unit of analysis in this paper refers to individuals.

Subjects with a high educational level completed the questionnaire themselves; interviewers later checked the responses in a face-to-face manner with the respondents. For subjects with low and medium educational levels, the data-collection method involved questionnaire-based face-to-face interviews. The response rate was 100%.

### Measurements

#### Questionnaire design

We used an omnibus questionnaire of 96 items that focused on themes of family functioning: economic, education, cohesion-solidarity, and sexual reproduction. Significance testing and anticipated model validation tests were based on Pearson chi-square statistics with 5–20 degrees of freedom. The use of this test imposes specific restrictions on sample size (optimum number, 900 subjects) and the Likert scales used (optimum number of responses, 3). The scale of violent behaviour appeared to have good internal consistency: Cronbach’s alpha = 0.95. All items correlated with the total scale to a good degree (lower r = 0.61). All items appeared to be worthy of retention: the greatest increase in alpha came from deleting the item that referred to threatening with a knife or a firearm, but removing this item increased the alpha by only 0.01. After conducting a 50-subject pilot survey, we created the final version of the questionnaire. A three-point Likert scale was used in the questionnaire to accommodate the sample size.

In the present study we focused on four items, namely:

a) During your childhood and/or adolescence: -Did you see or hear your parents hitting each other? -Were you beaten by your parents or other members of your family? Response options: Often, Occasionally, Never.

b) What feelings do you have when thinking of the family you grew up with? -Family is a refuge, a welcoming environment of affection. -Family is like a prison in which parents make the rules and do what they please with their children, modelling them as they see fit. -Family lets you handle things yourself, intervening only when problems occur. Response options: Yes, No.

c) How often did your partner express his anger, displaying the following types of behaviour? (For female respondents only.) How often did you express your anger against your partner with the following types of behaviour? (For male respondents only.)

(1) Insults, swearing, humiliation; (2) Withholding sexual activity or affection; 3) Slapping, kicking, pushing; (4) Hitting with a stick or other objects; 5) Threatening with a knife or firearm; 6) Being forced to engage in unwanted sexual intercourse; 7) Financial control, neglect, food deprivation. Response options: Often, Occasionally, Never.

d) Do you agree with the following statements about violence? -Violence is a necessary mark of strength and force. -A real man is not afraid to fight. -It is shameful to leave (to run from) a fight. -It is a natural response to use violence if someone tries to damage your property. -I see myself as a violent person.

-It is normal to hit a woman if you need to teach her a lesson. -I have the tendency to react physically first, and then use reason. -It is acceptable to hit somebody if they hit you first. -I like watching violence on TV, at the cinema, and like watching violent sports (e.g., boxing). -If I hurt people I feel bad afterwards and I even blame myself. Response options: Yes, No. Items from the Maudsley Violence Questionnaire [25].

A large number of articles as well as questions from various studies, surveys, and guides were used to create and modify the questionnaire, and the result was the four items indicated above [26-34]. Two conditions were taken into account when selecting items from the Maudsley Violence Questionnaire: a) two items were selected at each level of ‘machismo’ factor values of 0.5, 0.4, 0.3, and under 0.3 and b) items were selected that would be easy to understand within a Romanian cultural context.

### Ethical considerations

Written informed consent was obtained from each participant at recruitment. The subjects were informed that they could withdraw from the study at any stage and were assured of confidentiality. The study was approved by the Ethics Commission of the “Francisc I. Rainer” Anthropology Institute of the Romanian Academy.

We used the services of eight qualified interviewers to collect and check the responses; all the interviewers had expertise in the fields of sociology, psychology, and medicine. To ensure that the respondents felt at ease and to guarantee accurate answers, the respondents were assigned operators of the same gender and very broadly of similar age. Care and sensitivity were used at all times when dealing with the respondents.

With respect to a WHO recommendation that states that men and women should not be interviewed at the same time as it may put the woman at risk, a time delay of 6 to 10 months for each partner was used when surveying couples. The interviews were held in specially designated rooms: offices of family physicians, rooms made available with the help of village mayors, and university lecture rooms.

### Data management and statistical analysis

Demographic variables used in the statistical analyses were environment, gender, educational level, and age group. Statistical analysis, the Pearson chi-square test, and LCA were performed using the statistical programs SPSS (SPSS Inc., Chicago, IL) and Latent Gold (Statistical Innovations Inc., Belmont, MA). We employed LCA to examine distinct patterns of violence and beliefs about the use of violence among participants.

LCA is a statistical method for identifying unobservable classes (subgroups) within a sample by means of categorical or continuous observed variables and was preferred to the conventional linear regression (or log-linear) analysis that makes two restrictive assumptions about data that are often violated in practice: (1) the dependent variable is continuous with the normally distributed prediction error; and (2) the population is homogeneous—one model holds for all cases. The LCA associations between demographic covariates and class membership may be used to identify and explain the associated risk factors for an individual’s social attitudes and behaviour. The conditional probability indicates the chance that a member of a class to answer in a certain category (Yes or No) for the specific item. Conditional probability should be interpreted only by its value. Values close to 1 indicate higher chances. For an item, these probabilities sum to 1 within each cluster (column). The conditional probabilities characterize the latent classes.

The basic idea with an LCA is that parameters of a regression model differ across unobserved classes, and this offers a release from the restrictive assumptions of regression analysis. LCA uses observed categorical or binary data (or indicators) to identify homogeneous patterns, which are termed clusters or classes of a latent construct. In the present study, the observed data were binary responses (yes or no) to 10 items describing an individual’s behaviour, beliefs, or ways of thinking about violence. Individuals with similar response patterns were placed into the same violence class. We chose this procedure because the literature suggests that beliefs that legitimize violence and belief in the use of violence to enhance self-esteem are important predictors of violent behaviour [35].

The Bayesian Information Criterion (BIC) and Consistent Akaike Information Criterion (CAIC) statistics were used to evaluate the overall statistical model fit along with other fit statistics. A good model fit was generally indicated by low values of the BIC and CAIC statistics. Bivariate residuals (BVRs) provided a direct check of the LCA local independence assumption. When BVRs are quite large, this indicates that significant associations (local dependencies) exist among the variables.

Based on the BVR diagnostic statistic, two items of an initial 10 items (2: A real man is not afraid to fight and 5: I see myself as a violent person) were eliminated from the analysis. LCA was performed separately for the male and female subsamples.

## Results

### Magnitude and socio-demographic factors of domestic violence between parents and by parents against children

In all, 35% of the subjects witnessed violence between their parents in childhood or adolescence (6.9% often, 28.1% occasionally) and 53.7% were victims of family violence (5.6% often, 48.1% occasionally). In childhood or adolescence, 39.1% of all subjects had not witnessed violence between their parents and were not victims of family violence; 27.8% had witnessed violence between their parents and were victims of family violence; 25.9% had not witnessed violence between their parents but were victims of family violence; and 7.1% witnessed violence between their parents but were not victims of family violence. The proportion of subjects that witnessed violence between their parents during childhood and/or adolescence was greater in rural areas (40.1% in urban vs. 59.9% rural areas; chi-square = 18.427; p < 0.001), and respondents with a low level of education were more likely to have witnessed violence (49.7% in low-level education subjects vs.18.4% in high-level education subjects) in the FOO (chi-square = 35.515; p < 0.001). Victims of violence perpetrated by parents were more likely to be male (53.3% males vs. 46.7% females; chi-square = 13.048; p < 0.001). There were no statistically significant differences with respect to birth cohort regarding domestic violence between parents and by parents against children.

### Opinions related to the FOO and violence in childhood, violence against woman for corrective purposes

Most subjects regarded their FOO as ‘a refuge, a welcoming environment of affection’ (750 subjects, 86.3%); the second most strongly expressed opinion was that the ‘family lets you handle things on your own and intervenes only when difficulties occur’ (403 subjects, 46.4%). However, 6.7% declared that the ‘family is almost a prison, in which parents treat their children how they want, shaping them as they please’. There were no statistically significant differences in terms of environment, gender, educational level, or birth cohort regarding opinions relating to the family in which the subjects were raised. The proportion of subjects who perceived the family as almost like a prison (in which parents treated their children as they pleased, shaping them as they liked) was greater among those who witnessed domestic violence between their parents (chi-square = 9.317, p = 0.016) and among those who were physically beaten within their FOO (chi-square = 5.795, p < 0.005; Table 2).

**Table 2:** Violence in childhood and teenage years in the FOO and perceptions of the family home as an unwelcoming environment

**Violence in childhood or adolescence between parents, against children**		**Family home like a prison**^**a**^		**Total %**
		**No**	**Yes**	
Have you seen or heard your parents hitting each other?*	No	95.2	4.8	100
	Yes	89.8	10.2	100
Have you been hit, beaten by parents or other members of the family?**	No	95.5	4.5	100
	Yes	91.4	8.6	100

Legend: FOO, Family of origin, *p = 0.016, **p < 0.005, ^a^Family is almost a prison in which parents treat their children as they please, shaping them as they like.

The proportion of subjects who agreed with the statement that violence against woman was acceptable for corrective purposes was greater among those who had witnessed violence between their parents (chi-square = 12,730, p < 0.001) and among those who had been physically beaten within their FOO (chi-square = 22.901, p < 0.001; Table 3). The proportion of subjects who agreed with the statements ‘Family is almost a prison, in which parents treat their children how they want, shaping them as they please’ and ‘It is normal to hit a woman if you need to teach her a lesson’ was greatest among those who had often witnessed domestic violence between their parents and had often been beaten within their FOO.

**Table 3:** Violence in childhood and adolescence in the FOO and agreeing with violence for corrective purposes

**Violence in childhood or adolescence between parents, against children**		**It is normal to hit a woman if you need to teach her a lesson**		**Total**
		**No**	**Yes**	
Have you seen or heard your parents hitting each other?*	No	95.9	4.1	100
	Yes	89.8	10.2	100
Have you been hit or beaten by parents or other members of the family?*	No	98.0	2.0	100
	Yes	90.1	9.9	100

Legend: FOO, Family of origin, *p < 0.001.

### Magnitude and distribution of types of abuse against the wife or female partner in the FOP

Women were asked if they had been victim to any of seven types of violent behaviour (as described above in the section on questionnaire design) from their partners. Men were asked if they had engaged in any types of violent behaviour towards their partner. We obtained responses from 410 males and 444 females; 15 subjects did not respond, as they had never had a partner. The prevalence of domestic violence against women from their partner was 56.3%, which indicates that they had incurred at least one type of violent behaviour. Among the women who had suffered violence within the FOP, almost half had undergone more than two types of violent behaviour during the couple’s lifespan.

The most reported forms of abuse were insults, swearing, and humiliation, which were together identified by 45.1% of respondents: 392 subjects reported this type of violence, of whom 76 stated that this had occurred often and 316 that it had taken place occasionally. In terms of frequency, this form of abuse was followed by withholding sexual activity or affection (19.2%) and slapping, kicking, and pushing ((18.3%); Table 4).

**Table 4:** Prevalence of the seven manifestations of violence, against the wife or female partner in the FOP

**Manifestation of violent behaviour**	**Often**		**Occasionally**		**Total**	
	**Frequency**	**%**	**Frequency**	**%**	**Frequency**	**%**
Insults, swearing, humiliation	76	8.7	316	36.4	392	45.1
Withholding sexual activity or affection	26	3.0	141	16.2	167	19.2
Slapping, kicking, pushing	38	4.4	121	13.9	159	18.3
Hitting with a stick or other objects	24	2.8	32	3.7	56	6.4
Threatening with a knife or firearm	11	1.3	9	1.0	20	2.3
Being forced to engage in sexual intercourse or unwanted sexual fantasies	18	2.1	50	5.8	68	7.8
Financial control, neglect, food deprivation	16	1.8	33	3.8	49	5.6

Legend: FOP, Family of procreation.

The distribution of the manifestation of the seven types of violent behaviour is shown in Table 5. This behaviour was categorized as follows: emotional or psychological violence (1—insults, swearing, humiliation); physical violence (3—slapping, kicking, pushing; 4—hitting using a stick or other objects; 5—threatening with a knife or firearm); sexual violence (2—withholding sexual activity or affection; 6—being forced to engage in sexual intercourse or unwanted sexual fantasies); and financial abuse or neglect (deprivation) (7—financial control, neglect, food deprivation).

**Table 5:** Exposure of women to psychological, physical, sexual, and financial abuse or neglect from the intimate partner in the FOP

**Types of violence to which women were exposed**		**Male violent behaviour manifested towards women by gender**				
		**Violence by men reported by men**^**a**^		**Violence by men reported by women**^**b**^		**Violence by men**^**c**^
		**Frequency**	**%**	**Frequency**	**%**	**Total**
Emotional, psychological violence	Occasionally	159	50.30	157	49.70	316
	Often	32	42.1	44	57.9	76
Physical violence	Occasionally	73	33.83	89	66.17	162
	Often	25	33	48	67	73
Sexual violence	Occasionally	89	45.1	102	54.9	191
	Often	11	24.55	33	75.45	44
Financial abuse, neglect (deprivation)	Occasionally	10	30.30	23	69.70	33
	Often	7	43.80	9	56.30	16
Violent behaviour of men		406	44.56	505	55.44	911

Legend: FOP, Family of procreation,^ a^Violent behaviour by men against the partner or wife, reported by men, ^b^Violent behaviour by men against partner or wife, reported by that partner or the wife, ^c^Violent behaviour by men reported by men and women.

Negative results were higher among respondents living in rural areas than in residents of urban areas for the following factors: emotional (psychological) abuse (58.6% in rural areas vs. 33.1% in urban areas; chi-square = 56.110; p < 0.001) and physical abuse (33.4% in rural areas vs. 7.3% in urban areas; chi-square = 89.882; p < 0.001). The proportion for sexual abuse was higher for respondents living in urban than in rural areas: 27.9% vs. 18%, respectively (chi-square = 11.938; p < 0.001). Financial abuse or neglect (deprivation) was not influenced in a statistically significant manner by the living environment.

Negative results were more frequent among respondents with a low level of education than among those with a high level for the following factors: emotional (psychological) abuse (63.4% for low-level education subjects vs. 32.7% for in high-level education subjects; chi-square = 65.737; p < 0.001); physical abuse (37.2% for low-level education subjects vs. 8.6% for high-level education subjects; chi-square = 92.477; p < 0.001); and financial abuse or neglect (deprivation) (8.3% for low-level education subjects vs. 2.4% for high-level education subjects; chi-square = 8.874; p = 0.012). Sexual abuse was not influenced by the level of education in a statistically significant manner.

There was no significant statistical difference between the responses of males and females. Among women, 56.15% reported that they had been victims of emotional, physical, or sexual abuse, or controlling behaviour through their partners. Among men, 43.85% reported that they had engaged in some type of violence towards their partner. The prevalence of domestic violence was not significantly influenced by birth cohort. However, it was evident that there was a lower incidence among victims aged under 35 years than among older victims.

### Witnesses or victims of domestic violence in the FOO being transmitted to the FOP

Witnessing domestic violence between parents and being the victim of domestic violence in the FOO in childhood and adolescence was significantly related to all types of violence suffered by women and manifested by men in the FOP (p < 0.001). The highest proportion of all types of violence against women and exhibited by men in the FOP occurred among individuals who were both witnesses and victims of family violence in their childhood or adolescence in the FOO. In addition, the highest proportion of financial abuse and neglect (deprivation) in the FOP was observed among subjects who were both witnesses and victims of family violence in their childhood or adolescence in the FOO (Table 6).

**Table 6:** Types of violence to which women were exposed in the FOP and violence in childhood and adolescence in the FOO

**Types of violence to which women were exposed**		**Witnesses of family violence between parents and/or victims of family violence in childhood or adolescence**				**Total**
		**Not witnesses and not victims**	**Witnesses and victims**	**Not witnesses but victims**	**Witnesses but not victims**	
Emotional, psychological*	No	49.8	18.4	26.0	5.8	100
	Yes	26.5	39.0	25.5	8.9	100
Sexual*	No	43.2	25.7	23.9	7.3	100
	Yes	25.5	35.2	32.1	7.1	100
Physical*	No	43.7	22.9	27.2	6.2	100
	Yes	21.3	47.1	20.1	11.5	100
Financial abuse, neglect (deprivation)*	No	40.5	26.2	26.3	7.0	100
	Yes	16.3	55.1	16.3	12.2	100

Legend: FOO, Family of origin, FOP, Family of procreation, ^*^p < 0.001.

### Profile of beliefs and judgements regarding violent behaviour using LCA

Table 7 presents the LCA best-fit selection. In the male subsample, the best-fit model identified was that with three classes based on a BIC value close to the minimum (BIC = 3324.60) and lower bivariate residual values, which provided a good indication about compliance with the assumption of local independence in LCA. With LCA in the female subsample, the lowest values of the BIC (BIC = 2776) indicated the model with two classes as the best-model fit. Only two of the eight conditional probabilities of the indicator variables underlined the differences between the two classes (Table 7).

**Table 7:** Fit statistics for latent class models

	**Sample structure**			
	**Male only**		**Female only**	
**Model**				
	BIC	CAIC	BIC	CAIC
Cluster 1	3582.64	3590.64	2887.50	2895.50
Cluster 2	3321.18	3338.18	2776.62	2793.62
Cluster 3	3324.60	3350.60	2800.69	2826.69
Cluster 4	3360.80	3395.80	2843.20	2878.20
Cluster 5	3396.71	3440.71	2884.45	2928.45
Cluster 6	3439.44	3492.44	2927.50	2980.50

Table 8 shows beliefs and judgements about violent behaviour according to gender, environment, and history of violence in the FOO. The distribution of men within the three clusters was as follows: Cluster 1 (non-violent) accounted for 45% of males; Cluster 2 (moderately violent) 45%; and Cluster 3 (impulsive-reactive) 10%. Among males, the LCA model indicated a clear separation of the clusters for almost all the items, with the exception of the item referring to feelings of guilt after having hurt another person, in which no significant differences were observed. For seven of eight items, the observed conditional probabilities for a violent attitude increased from the non-violent cluster to the impulsive-reactive cluster. For example, the probability of an affirmative response to the item “It is OK (or normal) to hit women if you need to teach them a lesson” was only p = 0.01 in the non-violent cluster; it increased somewhat to p = 0.08 in the moderately violent cluster, but the increase in the impulsive-reactive cluster was up to p = 0.63.

**Table 8:** Profiles of beliefs and judgements about violent behaviour according to gender, environment, and history of violence in the FOO - Conditional probabilities

	**Male model**			**Female model**	
	**Cluster 1 non-violent**	**Cluster 2 moderately violent**	**Cluster 3 impulsive-reactive**	**Cluster 1 non-violent**	**Cluster 2 reactive**
Cluster size	45%	45%	10%	74%	26%
Indicators					
Violence is a sign of power and strength					
No	0.99	0.88	0.60	0.96	0.84
Yes	0.01	0.12	0.40	0.04	0.16
It is shameful to walk away from a fight					
No	0.89	0.48	0.29	0.86	0.55
Yes	0.11	0.52	0.71	0.14	0.45
It is OK to be violent if someone threatens to damage your property					
No	0.97	0.28	0.07	0.92	0.23
Yes	0.03	0.72	0.93	0.08	0.77
It is OK (or normal) to hit women if you need to teach them a lesson					
No	0.99	0.92	0.37	0.98	0.95
Yes	0.01	0.08	0.63	0.02	0.05
I tend just to react physically without thinking					
No	0.99	0.90	0.16	0.95	0.77
Yes	0.01	0.10	0.84	0.05	0.23
It is OK (or normal) to hit someone if they hit you first					
No	0.88	0.43	0.08	0.96	0.37
Yes	0.12	0.57	0.92	0.04	0.63
I enjoy watching violence on TV or in films, violent sports (e.g. boxing)					
No	0.84	0.65	0.26	0.95	0.81
Yes	0.16	0.35	0.74	0.05	0.19
When I have hurt people I feel bad or even hate myself for it afterwards					
No	0.34	0.22	0.32	0.26	0.22
Yes	0.66	0.78	0.68	0.74	0.78
**Covariates**					
Environment					
Urban	0.49	0.48	0.35	0.54	0.48
Rural	0.51	0.52	0.65	0.46	0.52
Witnesses of family violence between parents in childhood or adolescence					
No	0.72	0.62	0.36	0.68	0.60
Yes	0.28	0.38	0.64	0.32	0.40
Victims of family violence in childhood or adolescence					
No	0.43	0.40	0.22	0.55	0.44
Yes	0.57	0.60	0.78	0.45	0.56

Legend: FOO, Family of origin.

The distribution of women within the two clusters was as follows: Cluster 1 (non-violent) accounted for 74% of women; Cluster 2 (reactive) 26%. In the non-violent cluster, any violent behaviour was very unlikely. In the reactive cluster, there was a higher probability of accepting violent behaviour in situations when someone hit the individual first (p = 0.63 vs. p = 0.04) or threatened to damage their property (p = 0.77 vs. p = 0.08) (Table 8).

The distribution of subjects across the latent classes indicated probabilistic differences explained by the covariate variables: witnesses of family violence between parents and victims of family violence in childhood or adolescence. Among males, there was a greater likelihood of those who witnessed parental violence during their childhood being in the impulsive-reactive Cluster 3: p = 0.64 (conditional probability that the subject witnessed parental violence as a child) vs. p = 0.36 (conditional probability that the subject did not witness parental violence). In addition, the impulsive-reactive cluster was more likely to include men who were victims of violence during childhood: p = 0.78 vs. p = 0.22. There was a greater incidence of men in rural areas being in the impulsive-reactive cluster: p = 0.65 vs. p = 0.35.

Among women, there was a greater likelihood of those who were victims of violence during childhood being in the reactive Cluster 2: p = 0.56 vs. p = 0.44. There was a greater representation among women in rural areas in the reactive cluster: p = 0.52 vs. p = 0.48.

There were no statistically significant differences by birth cohort with respect to beliefs and judgements about violent behaviour.

## Discussion

Methods for collecting data and classifying statistics on domestic violence are not uniform worldwide; it is therefore impossible to conduct an accurate comparison among countries. Comparing data on domestic violence among countries is also difficult owing to cultural differences and perceptions of the concept of abuse from one country to another. Nevertheless, we can make same comparisons between the present study and other studies, especially Romanian studies, which have used similar methodologies for data collection and questionnaire surveys.

### Magnitude and socio-demographic factors of domestic violence between parents and by parents against children in the FOO

In the entire sample, during childhood and adolescence in the FOO, more than a quarter of the subjects had seen or heard their parents striking each other, and more than half had been beaten by their parents or other family members. The frequency of such violent events was reported as ‘often’ by 12.5% of subjects. More than a quarter of the subjects were both witnesses and victims of these violent events in the FOO. In rural areas, there was a higher proportion of witnesses among subjects with a low education level; in both urban and rural areas, a greater percentage of the victims of family violence were boys in the FOO.

Studies indicate that there is increased risk when violence is considered a natural response or its use is acceptable or justified in ‘correcting’ other people [36].

This particular relationship between parents and children is evident in the comment ‘I gave life to you, and so I can take it away’ and in the proverbs ‘When you beat the mother, the baby grows’ and ‘Beat the child to make him a man’; such comments and sayings are frequently used by Romanian parents for corrective purposes.

In the Romanian National Study SSR-Ro 2004, approximately 20% of the 4,441 women had witnessed violence between their parents or been subjected to violence by family members during childhood or adolescence. Of the 2,361 men in that study, about 20% had witnessed violence between their parents, and 66.7% had been subjected to violence by family members during childhood or adolescence [16]. In the present study, there was a lower proportion of men who had been physically abused by their parents or other family members than in the 2004 study. The present study found a greater proportion of physical abuse (as witness or victim) in rural areas and among those with a lower educational level than in the 2004 study. The present study identified a similar prevalence of violence in the FOO to that reported in a study by Rada and Tarcea, which was conducted from 2005 to 2006 on 941 men and 961 women aged 15–90 years and living in urban areas of Romania [37].

The large number of children who were beaten in their FOO is a matter that necessitates educational intervention in Romania. It is essential for educational programmes to clearly state the counterproductiveness of using force in dialogues between partners in an attempt to subordinate the other party and to indicate the risk of entering a cycle of generational violence. It is important to stress in such programmes that striking children does not teach them to realize what is wrong with their behaviour.

In Romania, using beatings as a form of corrective behaviour and the projection of personal deficiencies and frustrations into violence towards children remain a serious problem; in such cases, the intervention required is of a psychological-educational nature. A major problem here is the suspension of psychological services and the prohibition of psychological interventions during the communist era. Despite the recent developments in social assistance since 1990, there is a low acceptance in the public and private sectors for personal development and psychological interventions.

### Opinions related to the FOO and violence in childhood and violence against woman for corrective purposes

Over 90% of the subjects regarded their FOO as ‘a refuge, a welcoming environment of affection’ and held the belief that the ‘family lets you handle things on your own and intervenes only when difficulties occur’. The proportion of subjects who perceived the family as an unfriendly environment and of those who agreed with violence against woman for corrective purposes was over two-fold among those that had witnessed domestic violence between their parents and those that were beaten within their FOO.

Further clarification is required to explain the significant difference between the high number of subjects who witnessed violence between their parents in childhood or adolescence (over one-quarter) and who were beaten (over half) and the belief of over three-quarters of the subjects that the FOO was a welcoming environment (more than three-quarters); it would seem that the opposite should be true. It is possible that the subjects internalized the idea that education or correction was achieved through violence and thus continued to see their FOO as a haven. Furthermore, for many subjects, the inter-parental violence that they were asked to consider occurred a long time in the past. If someone witnessed or was a victim of parental violence during childhood and the relationship between or with parents later improved, that may have reduced negative perceptions of the FOO. We should also take into consideration the psychological loyalties towards the FOO—loyalties that often grow stronger when the parents pass away [38]. Previous studies have also found that while there is a significant negative relationship between marital satisfaction or conflict and IPV, most respondents nevertheless stated that their relationship was a good one [39].

Another explanation for positive feelings towards the FOO among those who were beaten in the family could be the following. It is naturally assumed that almost all parents love their children. When parents punish them by beating, children may think that love and violence go together [40].

### Magnitude and distribution of types of abuse against the wife or female partner in the FOP

Different studies have found that the prevalence of various forms of IPV varies during a person’s lifespan. Recent international studies have estimated that 24%–43% of people experience IPV in their lifetimes. Nevertheless, in countries with a low level of development or a patriarchal model of perception of gender roles, the prevalence of IPV is greater [41-45].

In the present study, controlling behaviour or emotional, physical, or sexual abuse against women by the partner was expressed as having occurred at least once in more than half of the cases. The prevalence of IPV against women found in the present study is comparable with that reported for Mexico [42,46].

In the Romanian national survey SSR-Ro 2004, at least one of emotional, physical, or sexual abuse against women by the partner was reported in over 30% of cases. The Rada-Tarcea study in 2005–06 on violence against women by their partners also found that at least one type of violence (controlling behaviour or emotional, physical, or sexual abuse) was reported in over 30% of the cases. In the present study, the rates were approximately 19% higher.

Since these three Romanian studies used similar questions and methods of data collection, it is possible that the higher prevalence in the present study was due to the sample population. The 2004 study may have underestimated the true prevalence in the population: for psychological reasons, some women feel too ashamed or scared to report abuse and therefore may have understated or failed to mention aggressive acts despite the fact that they were assured of complete confidentiality. In addition, Romanian men previously tended not to recognize sexual abuse because they were simply unaware of the concept: at one time, the man believed that his female partner was obliged to have sex with him whenever he wanted.

The Rada-Tarcea study was conducted only in urban areas, and there is generally a lower prevalence of domestic violence in such areas in Romania. This could explain the lower prevalence of violence against the partner found in that study than in the present one, which included subjects from rural areas. The higher rates of violence reported in the present study could also be the result of empowerment—an awareness of the meaning of real domestic violence; this could have resulted in increased courage to mention acts of aggression and greater knowledge about the definition of domestic violence in relation to human rights. But it is of course also possible that there was a real increase in domestic violence or a higher prevalence in the regions examined in the present study. These issues require further examination. Unfortunately, the last national study in Romania that included domestic violence was conducted in 2004. In the present study, the fact that the violence suffered and reported by women correlated with that perpetrated and reported by men suggests that the responses given in the questionnaire could be honest and accurate.

High levels of psychological abuse have also been recorded in other studies [46,47]. The fact that the present study shows a higher proportion of psychological abuse than other types of violence indicates the need for prevention and intervention measures with this type of IPV. In addition, psychological abuse almost always accompanies other types of violence and precedes physical abuse. It is often easier to record physical violence than psychological and sexual abuse since physical abuse is frequently accompanied by official evidence (e.g., doctor’s and police reports) [39]. Women’s health may be affected by both psychological and physical abuse. This indicates the need to develop sensitive interventions for psychological abuse as well as for physical abuse, which receives greater attention since it tends to be considered ‘out in the open’. Family doctors also have to check whether IVP symptoms stem from psychological abuse, which is harder to identify. In addition, there is a need for health professionals to receive training on domestic violence issues [48,49].

The majority of data presented in other studies relates to physical violence. In the present study, physical abuse against women by the partner was expressed in around a quarter of the cases. The prevalence of physical abuse in this Romanian sample is comparable with that in developed countries in Europe and elsewhere [50,51]. In addition, sexual abuse was expressed in around a quarter of the cases in the present study. The prevalence of physical and sexual abuse against women in this Romanian sample was about half the average psychological and sexual abuse during a couple’s lifespan in 15 sites in Bangladesh, Brazil, Ethiopia, Japan, Peru, Namibia, Samoa, Serbia and Montenegro, Tanzania, and Thailand [52].

It is also notable that half of all violence experienced by the women in the present study involved more than two types of violent behaviour. The proportion of both emotional (psychological) abuse and physical abuse was on average over two-fold higher among respondents who lived in rural areas and those with a low level of education. Similarly, financial abuse and neglect (deprivation) was over three times more frequent among those with a low level of education. Interestingly, sexual abuse was reported 1.55 times more frequently by subjects living in urban areas. All these demographic characteristics can be considered risk factors for violence against women by her partner.

In this study, a significant correlation was found between level of education and income. Previous studies have found that differences in income and differences in education between partners are considered risk factors for juvenile delinquency, violence, and criminality [53]. In the present study partners had similar levels of education and income. The differences between education and income status were not significant factors that influenced violence between partners. Therefore, the data in our study do not support the claim that men with lower levels of education than their partners are more likely to be violent towards those partners [54].

There is also considerable evidence that the rate of domestic violence decreases as couples age [55-57]. In this study, differences in reports of violence within couples before the age of 34 years and those afterwards could have the duration of exposure as a confounding factor: older women had a longer exposure period, and older men had the opportunity to commit abuses over a longer period of time. The association between intimate partner violence and age is better reflected in recent studies that have assessed abuse over shorter periods such as the previous 12 months.

### Extent to which being a witness or victim of domestic violence in the FOO is transmitted to the FOP

Like other studies, the present one identifies a correlation between physical abuse in childhood and violence against a woman by her male partner [58]. Our study indicates a correlation between experiencing abuse during childhood (directly or indirectly) and then directing anger towards one’s intimate partner or accepting such violence as natural.

In the correlation between violence in the FOO and that in the FOP, the greatest manifestation of violence in the FOP was reported by subjects who were both witnesses and victims of violence in the FOO. Those who both did not witness domestic violence between their parents and were not victims of domestic violence in the FOO in childhood and adolescence reported the highest absence of violence in the FOP. It is noteworthy that subjects who were victims of family violence more frequently reported emotional (psychological), sexual, or physical abuse than those who only witnessed violence between their parents. In this context, it is necessary to consider that adults who were victims of physical abuse as children are more likely to be perpetrators of physical abuse to children as adults [59,60].

### Profiles of beliefs and judgements about violent behaviour according to gender, environment, and history of violence in the FOO using LCA

Intimate partner violence was seen as having roots in gender inequality or as a response to the conflicts generated by increasing gender equality and women's attempts to gain autonomy [61].

Some studies have suggested that gender differences with respect to aggression are a function of perceived consequences of aggression that are learned as aspects of gender and other social roles [62]. Using items from the Maudsley Violence Questionnaire [25] to measure perceptions relating to violent behaviour, this study made the following findings. We found the proportion of violent beliefs and judgments among men to be almost 30% higher than among women. In addition, LCA identified an impulsive-reactive cluster in men, which registered the highest probability for pro-violent beliefs and judgments. We were unable to establish such a cluster in women. The impulsive-reactive cluster, which was characterized by pro-violent beliefs and judgments, predominantly consisted of men who had witnessed parental violence and were victims of violence. Women who were victims of violence during childhood tended to appear in the reactive cluster. In this cluster, there was a high probability only for accepting violent behaviour in situations when someone hit the individual first or threatened to damage their property. There was a higher prevalence of the impulsive-reactive cluster for men and the reactive cluster for women in rural areas.

Walker and Bowes consider that violent thinking (specifically machismo types) should be included in assessments of violent offenders. Work on violent thinking and reducing machismo thinking could be a useful adjunct in anger management with violent offenders [63].

In the present study, the correlations among beliefs, pro-violent thoughts, and violence against partners and children in the family were examined in terms of the education of children and adolescents with respect to the development of pro-social behaviour. An aggressive climate within the family and outside may raise aggression in the short term by increasing aggressive thoughts. These results are important in understanding the long-term effects of violence for both witnesses and victims. Changing behaviour starts with changing beliefs and counterproductive thoughts.

## Conclusions

Like other studies, the present one has identified the following risk factors for violence in the FOP: living in a rural area, a low level of education, being female, and coming from a family in which violent behaviour between parents and against children occurred. Thus, in the context of Romania, it is necessary to establish preventive measures to assist people living in rural areas, especially women [64-66].

Comparing the results of studies on domestic violence made between 2003 and 2007 in Romania with the present study findings, we conclude that the prevalence of IPV against women and family violence on children has not declined. Despite efforts to improve legislation regarding IPV, the implementation of governmental and nongovernmental programs, and the actions of various scientific and artistic personalities in media campaigns against violence, the prevalence is still high. Attitudinal and behavioural patterns have a high resistance to change, and the phenomenon of violence in Romania has its roots in the communist period.

Communism in Romania was created based on a traditional patriarchy—state patriarchy. On the one hand, communism proclaimed gender egalitarianism, but it also insidiously produced and maintained a new inequality between men and women. Studies have suggested that, especially in nonindustrial societies, some activities contribute to the sex-typed division of labour and patriarchy. Biosocial analysis suggests that differences in the behaviour of women and men derive from the interaction between the physical specialization of the sexes [67].

In general Romanian society, particularly in families, boys learn dominance and girls submission. Within families—especially those from the lower and middle classes—women have limited freedom. If a woman expresses her opinion, this is regarded as nagging and argumentative behaviour [68,69].

The use of beatings as a form of corrective behaviour remains a serious problem in Romania. Lower income levels in rural areas hinder educational efforts to present alternatives to violence. Furthermore, at a national level, the funding set aside for such interventions is insufficient. Therefore, when designing IVP policies, the Romanian government needs to focus on its vulnerable populations and increase assistance in rural areas.

### Limitations of the study

This study does have some limitations, and they include the following. The size of the sample decreased the relevance for detecting associations by multivariate analysis. No data were collected regarding other risk factors of violence within families, such as alcohol or drug abuse, inability to resolve social problems, poor parenting practices and family functioning, negative peer influence, access to firearms, and neighbourhood. This study did not include a representative sample, and we are therefore unable to generalize our results.

Pilot surveys and previous research have shown that subjects with low and medium educational levels, especially in rural areas, generally do not have the ability to complete questionnaires, so self completion of the questionnaires was not an option. Most subjects with high education levels have commented: ‘We do not need someone to do the interview with us; we know better how to fill [out the questionnaire]’. Therefore, we had to give up questionnaire-based face-to-face interviews with such subjects. These two different ways of collecting data (questionnaire-based face-to-face interviews for subjects with low and medium educational levels and self-completion for subjects with a high educational level) are likely to have introduced a bias. However, since interviewers later checked the responses in a face-to-face manner with the respondents, the bias may be a not significant one.

Regarding the questions on the seven types of violent behaviour (classified by type of violence), defining the quantitative measurements in terms of frequency—‘often’ and ‘occasionally’—gave room for subjective interpretation: such intensities may have been perceived differently.

In the case of domestic violence, the problem is generally more a quantitative one. Using the case of smoking cigarettes as an analogy, there is no question that the number of cigarettes smoked daily is correlated with the risk of lung cancer. The number of times a person is assaulted can be likened to the number of cigarettes smoked. However, one incident of sexual abuse or being threatened with a gun can have a long-lasting negative effect on a person’s life but one cigarette cannot. The victims simply feel the pain. With such questions, personal experience can result in differing interpretations of reporting abuse.

This issue also involves the issue of tolerance. Scales used in this study are commonly employed in such areas as clinical psychology, sociology, marketing, and medicine without any major problems of bias [70]. Moreover, regarding the intensity of violent behaviour in a couple’s relationship in the FOP with respect to questionnaire responses, the four qualified interviewers checked those responses in terms of the following categories: ‘constantly’ (Latin-derived) = ‘all the time’ (Germanic), ‘frequently’ (Latin) = ‘often’ (Germanic), ‘occasionally’ (Latin) = ‘sometimes’ (Germanic). Interviewers were also able to note on the questionnaire or in a notebook particular circumstances.

To reduce any bias based on the imprecision of ‘often’ and ‘occasionally’, in Tables 6, 7 and 8, statistical analyses have been performed with the dummy dichotomous variables of YES and NO, eliminating any degrees of intensity. The dummy variables were obtained by recoding the original ordinal variables into the binary format of 1 (YES) and 0 (NO). The dummy variable YES substituted for Often and Occasionally, and NO was a recoding for Never.

## Abbreviations

IPV: Intimate partner violence; FOP: Family of procreation; FOO: Family of origin; SSR-Ro 2004: Reproductive Health Survey Romania 2004; LCA: Latent class analysis; BIC: Bayesian Information Criteria; CAIC: Consistent akaike information criteria; BVRs: Bivariate residuals.

## Competing interests

The author declares that she has no competing interests.

## Authors’ information

CR (BA in psycho-sociology and PhD in medicine) is a scientific researcher at the “Francisc I. Rainer” Institute of Anthropology, Romanian Academy, Biomedical Department and is an Associate Professor at the “Spiru Haret” University, Faculty of Sociology-Psychology; she teaches Psychology of Families and Couples and Social Psychology. She is also a clinical psychologist and works in private practice. CR has accumulated experience from extensive research in the areas of sexual reproductive health and family health.

## Pre-publication history

The pre-publication history for this paper can be accessed here:

http://www.biomedcentral.com/1471-2458/14/129/prepub
